# The Antioxidant from Ethanolic Extract of *Rosa cymosa* Fruits Activates Phosphatase and Tensin Homolog In Vitro and In Vivo: A New Insight on Its Antileukemic Effect

**DOI:** 10.3390/ijms20081935

**Published:** 2019-04-19

**Authors:** Kuan-Chih Wang, Yi-Chang Liu, Mohamed El-Shazly, Shou-Ping Shih, Ying-Chi Du, Yu-Ming Hsu, Hung-Yu Lin, Yu-Cheng Chen, Yang-Chang Wu, Shyh-Chyun Yang, Mei-Chin Lu

**Affiliations:** 1 School of Pharmacy, Kaohsiung Medical University, Kaohsiung 807, Taiwan; dx9901mk5@gmail.com; 2 Division of Hematology-Oncology, Department of Internal Medicine, Kaohsiung Medical University Hospital, Kaohsiung 807, Taiwan; ycliu@cc.kmu.edu.tw; 3 Department of Internal Medicine, Faculty of Medicine, College of Medicine, Kaohsiung Medical University, Kaohsiung 807, Taiwan; 4 Department of Pharmacognosy and Natural Products Chemistry, Faculty of Pharmacy, Ain-Shams University, Organization of African Unity Street, Abassia, Cairo 11566, Egypt; mohamed.elshazly@pharma.asu.edu.eg; 5 Department of Pharmaceutical Biology, Faculty of Pharmacy and Biotechnology, German University in Cairo, Cairo 11835, Egypt; 6 Doctoral Degree Program in Marine Biotechnology, National Sun Yat-Sen University, 70 Lien-Hai Road, Kaohsiung 80424, Taiwan; m6430005@hotmail.com; 7 Doctoral Degree Program in Marine Biotechnology, Academia Sinica, 128 Academia Road, Section 2, Nankang, Taipei 11529, Taiwan; 8 Graduate Institute of Marine Biotechnology, National Dong Hwa University, Pingtung 944, Taiwan; ycdu0626@gmail.com; 9 National Museum of Marine Biology & Aquarium, Pingtung 944, Taiwan; 10 Research Center for Environmental Medicine, Kaohsiung Medical University, Kaohsiung 807, Taiwan; maiz538272@gmail.com; 11 Division of Urology, Department of Surgery, E-Da Cancer Hospital/I-SHOU University, Kaohsiung 824, Taiwan; ed100464@edah.org.tw; 12 College of Medicine, I-SHOU university, Kaohsiung 824, Taiwan; 13 The Ph.D. Program for Cancer Biology and Drug Discovery, China Medical University and Academia, Sinica, Taichung 404, Taiwan; j520c@hotmail.com

**Keywords:** anticancer, apoptosis, leukemia, phosphatase and tensin homolog, PTEN, *Rosa cymosa* Tratt

## Abstract

*Rosa cymosa* Tratt is a Chinese herbal remedy that is used in the treatment of diarrhea, burns, rheumatoid arthritis, and hemorrhage. Despite its use in Asian folk medicine, there are limited reports on the biological activity of *R. cymosa* fruits. This study focused on the investigation of the antitumor effect of the antioxidative ethanolic extract of *R. cymosa* fruits (RCE) along with its underlying mechanism of action. RCE showed a potent cytotoxic effect against Sup-T1 and Molt-4 lymphoblastic leukemia cells. In the xenograft animal model, the tumor size was significantly reduced to about 59.42% in the RCE-treated group in comparison with the control group. The use of RCE (37.5, 75, or 150 μg/mL) triggered apoptosis by 26.52–83.49%, disrupted mitochondrial membrane potential (MMP) by 10.44–58.60%, and promoted calcium release by 1.29-, 1.44-, and 1.71-fold compared with the control group. The extract induced redox oxygen species (ROS) generation through the elimination of Nrf2/Keap1/P62-mediated oxidative stress response. The loss of phosphatase and tensin homolog (PTEN) activation by RCE impaired PI3K/Akt/Foxo and Jak/Stat activation pathways, which contributed to tumorigenesis. These multiple targets of *R. cymosa* against hematologic cancer cells suggested its potential application as an antileukemic dietary supplement.

## 1. Introduction

The number of new adult cases of leukemia in the United States of America is approximately 13.8 per 100,000 persons per year [1,2]. Among these cases, the number of acute lymphoblastic leukemia (ALL) in adults is approximately 1.7 per 100,000 persons per year, with an estimate of 5960 new cases and 1470 deaths in 2018. The current first line of defense for adults suffering from ALL consists of a standard high dose of chemotherapy. However, the outcomes in adult ALL patients are generally poor, with overall survival rates less than 45% despite the introduction of late intensification and maintenance chemotherapeutic protocols as well as the use of the allogeneic hematopoietic stem cell transplantation technique especially in high-risk patients. Treatment of elderly ALL patients, which represent one-third of all patients, remains a critical issue with low success rates [3]. Intensive chemotherapeutic protocols are often associated with a high mortality rate, especially in elderly patients. ALL could be categorized into T-cell ALL (T-ALL) or B-cell ALL (B-ALL) based on the immunophenotyping of lymphoblasts. In recent years, considerable progress has been achieved in the treatment of B-ALL by targeting cell surface antigens on B lymphoblasts, such as CD20- and CD22-targeted monoclonal antibodies, bispecific T cell engaging (BiTE) antibodies, and chimeric antigen receptor (CAR) T cells [1,4,5,6,7,8,9,10]. On the other hand, the development of new agents for T-ALL has not undergone similar developments. Many patients of T-ALL suffer from relapse after achieving remission, and the treatment outcomes remain poor even after salvage therapy. Alemtuzumab is an anti-CD52 monoclonal antibody, which was investigated in CD52-expressing T-ALL and showed interesting results. However, the development is slow due to its modest activity and significant toxicity [10]. Nelarabine was found to be helpful in relapsed T-ALL and the results prompted its use in combination with other chemotherapeutic agents [11,12]. The NOTCH1 signaling pathway is an attractive target in T-ALL patients, and several small-molecule gamma-secretase inhibitors are currently being investigated in clinical trials [13]. There is clearly an unmet medical need for the development of new agents for the treatment of T-ALL patients. One of the most important steps for the development of new therapeutic agents to treat T-ALL patients is to understand the mechanisms of leukemogenesis and chemoresistance.

Emerging evidence has demonstrated that Raf/MEK/ERK, PI3K/PTEN/Akt/mTOR, and Jak/STAT pathways play important roles in inhibiting apoptosis in hematopoietic cancer cells [14,15,16,17]. Based on the gene expression profiling studies, the aberrant gene expression of oncogenic transcription is combined with the inactivation of tumor suppressor gene phosphatase and the activation of phosphatase and tensin homolog (PTEN) of the Notch1 pathway in T-cell transformation [18]. The tumor suppressor gene, PTEN, is a key regulator involved in cancer development, which suppresses the PI3K/AKT pathway that governs cell proliferation, growth, migration, energy metabolism, and death [19,20]. This gene is often inactivated by mutations (including deletions) in different cancers, especially in T-ALL patients. Therefore, the disruption of these signaling pathways may act as a promising target for leukemia therapy, especially in acute lymphoblastic leukemia.

*Rosa cymosa* Tratt (Xiao-jin-ying) is a deciduous climbing shrub with hooked prickle branches reaching 5 m in height [21]. It is a traditional Chinese herbal remedy that is used to relieve pain and treat burns. The roots are used in the treatment of diarrhea, rheumatoid arthritis, descensus uteri, and hemorrhage [22]. *R. cymosa* is an interesting shrub from several points of view because it can grow under harsh environmental conditions, such as in arid and dry lands. It exhibits a high tolerance to drought stress, which is an important characteristic feature of medicinal herbs especially with the recent changes in climate and the global elevation in temperature. This shrub can dominate a large area of land and become an important source of biologically active constituents [21]. Several secondary metabolites have been isolated from *R. cymose*, including pomolic acid, fupenzic acid, ursolic acid, euscaphic acid, arjunic acid, tormentic acid, 3β-E-feruloyl corosolic acid, 1β-hydroxyeuscaphic acid, myrianthic acid, cecropiacic acid, ilexoside B, cymosic acid 3β,19α-dihydroxy-2-oxo-12-ursen-28-oic acid, 2α,19α-dihydroxy-3-oxo-12-ursen-28-oic acid, ursane-type triterpenes, and terpenoid glycosides [23,24,25].

Ursane-type triterpenes showed a wide range of pharmacological effects such as hypoglycemic, hepatoprotective, antioxidant, immunomodulatory, anti-inflammatory, and antitumor activities without significant side effects even at higher concentrations [26,27,28]. Recent studies indicated that the ursane-type pentacyclic triterpenoids can act as a useful platform for the discovery of new anticancer drugs [29]. Despite the richness of *R. cymosa* in triterpenoids, its antitumor effect has not been fully investigated. A search in PubMed (Nov 2018) using the search term “*R. cymosa*” retrieved two peer-reviewed publications reporting the isolation of fourteen natural products from this plant. To unearth *R. cymosa*’s therapeutic potential, its antitumor effect along with its mechanism of action were examined in in vitro and in vivo models using lymphoblastic leukemia and lymphoma cells.

## 2. Results

### 2.1. Antitumor Effect of the Ethanolic Extract of R. cymosa (RCE)

**Figure 1:**
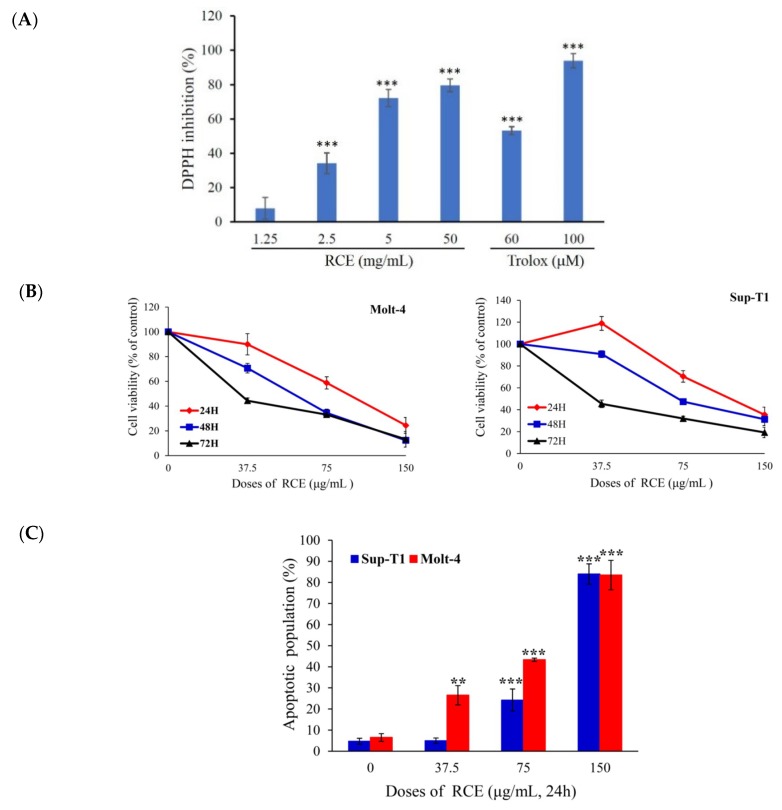
Effect of extract of *Rosa cymosa* (RCE) on cellular viability and apoptosis induction in the in vitro assay. (**A**) The anti-oxidative activity was determined by DPPH assay and Trolox served as the positive control; (**B**) Human leukemia Molt-4 and Sup-T1 cells were treated with RCE at different doses for 24, 48, and 72 h. Cell variability was determined by the MTT assay; (**C**) The apoptotic populations of Sup-T1 and Molt-4 cells were determined with flow cytometric analysis after treatment with RCE. Data are expressed as mean ± SD. * *p* < 0.05; ** *p* < 0.01; and *** *p* < 0.001 control vs. RCE group. The IC_50_ value was calculated using CalcuSyn software.

The morphology of *R. cymosa* fruits is shown in Figure S1. An electric mill was used to grind the dried fruits (700 g) into a fine powder. The powder was extracted exhaustively with ethanol (5 × 1 L), which was then lyophilized and stored at −70 °C (RCE). The antioxidant activity of RCE was evaluated using DPPH assay. RCE inhibited the DPPH formation in a dose-dependent manner with the EC_50_ of 3.02 mg/mL (Figure 1A). Although previous studies reported the cytotoxic activity of ursane-type triterpenes, the effect of RCE on cancer cell proliferation was never investigated. In the current report, we evaluated for the first time the effect of RCE on the cellular proliferation of two cancer cell lines, including Molt-4 and Sup-T1 cells, at different doses using MTT assay. The growth of Molt-4 and Sup-T1 cells was suppressed in dose- and time-dependent manners with IC_50_ values of 88.8 and 114.8 μg/mL after 24 h; 56.0 and 73.0 μg/mL after 48 h; and 34.23 and 31.15 μg/mL after 72 h, following the exposure to RCE at 37.5, 75, and 150 μg/mL, respectively (Figure 1B). The quantification of tetrazolium dye incorporation confirmed that the exposure to 150 µg/mL of RCE resulted in a significant reduction of cellular viability up to 24.38% and 38.38% in Molt-4 and Sup-T1 cells, respectively, after 24 h. Next, we investigated if apoptosis was involved in the cytotoxic effect of RCE on cancer cells. Annexin-V/PI staining showed that RCE treatment induced apoptosis in Molt-4 and Sup-T1 cells. The use of RCE (37.5 and 75 μg/mL) for 24 h increased the percentage of apoptotic cells from 4.96% to 24.24% in Sup-T1 and from 24.52% to 43.35% in Molt-4 cells compared with the solvent control. Interestingly, the percentage of apoptotic cells approached 90% with 150 µg/mL of RCE in both cell lines (Figure 1C). Thus, it may be concluded that Molt-4 cells were more susceptible to RCE cytotoxic effect compared with Sup-T1 cells.

**Figure 2:**
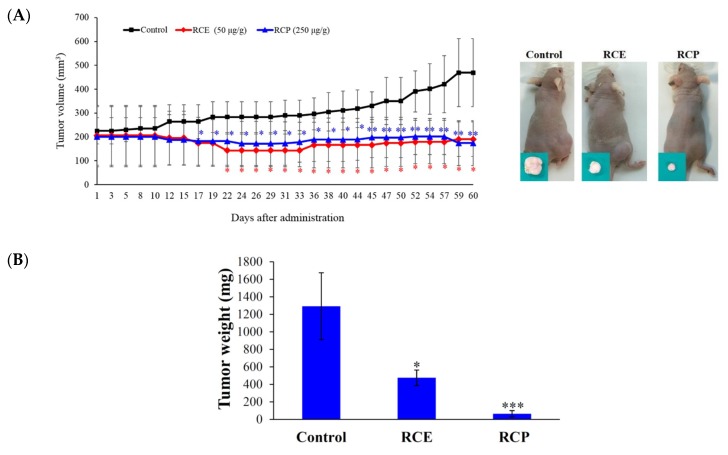
Effect of RCE on tumor growth in the in vivo xenograft animal model. (**A**) RCE and fruit powder of *R. cymosa* (RCP) inhibited tumor growth in the in vivo xenograft animal model inoculated with human leukemia Molt-4 xenograft. Male nude mice bearing leukemia Molt-4 tumors were treated with the solvent (negative control, *n* = 10), RCE (50 μg/g, *n* = 10), or RCP (250 μg/g, *n* = 10) for 60 days. Tumor volumes were measured every other day, and the results are expressed as mean ± SD. ** Significantly different from the control groups at *p* = 0.0053. Representative photos of subcutaneous tumors, which were collected after treatment with the solvent only (right), with RCE (middle), or RCP (left) after 60 days; (**B**) Histogram of the tumor weight from the control group and RCE-treated group. Values are expressed as the mean ± SD. Significantly different from the control group at * *p* < 0.05; *** *p* < 0.001.

The interesting cytotoxic effect of RCE against Molt-4 and Sup-T1 cells in MTT and flow cytometric assays encouraged us to study its effect on tumor formation in the animal model. The in vivo antitumor activity of RCE and fruit powder (RCP) was examined by studying their antitumor effect in xenograft animal models using human leukemia Molt-4 cells. Male immunodeficient athymic mice were injected subcutaneously at their right flank with Molt-4 cells (1 × 10^5^). The doses were established based on the cytotoxicity study of RCE causing 50% inhibition of cell proliferation after 72 h; therefore, we used 50 mg/kg of RCE in the in vivo experiment. RCP (700 g) was refluxed with ethanol in a 1:10 (*w*/*v*) ratio to yield 140 g of RCE (20% of RCP). Therefore, RCP was used at 250 mg/kg to examine its antitumor effect. The oral administration of RCP (250 mg/kg of body weight) and RCE (50 mg/kg of body weight) significantly suppressed tumor growth after 17 and 22 days of treatment, respectively. After 60 days of treatment, the average tumor volume in the control group was 469.04 ± 142.56 mm^3^, whereas the average tumor volume in the RCE- or RCP-treated group was 190.32 ± 72.19 and 174.20 ± 94.18 mm^3^, respectively (Figure 2A). The tumor volume was significantly reduced in the RCE and RCP groups by 59.42% and 62.86% as compared with the control group (*p* = 0.0053), respectively. No significant differences were observed in the mice body weights as well as in the histology of the heart, kidney, liver, lung, and spleen (Figures S2 and S3). In the control and RCE groups, tumor cells appeared as single oval round, high nucleic/cytoplasm ratio cells and they were highly mitotic (arrow). Smaller tumor cells showing less survival rate, focal necrosis, and cystic formation in the central area were found in the RCP group (Figure S3). The tumor tissue was isolated and weighed at the end of the treatment revealing smaller average weights in the RCE- and RCP-treated groups (474.9 ± 88.5 and 63.8 ± 35.4 mg) compared with the control group (1292.31 ± 382.1 mg) (Figure 2B). A reduction in tumor volume was observed with no significant changes in mice weights. In addition, no histological differences in mice tissues were detected, suggesting that RCE and RCP exhibited a potent anti-tumorigenic effect in the in vivo xenograft model without significant side effects.

### 2.2. Apoptotic Effect of RCE Is Mediated through Mitochondrial Dysfunction and Redox Oxygen Species (ROS) Generation

**Figure 3:**
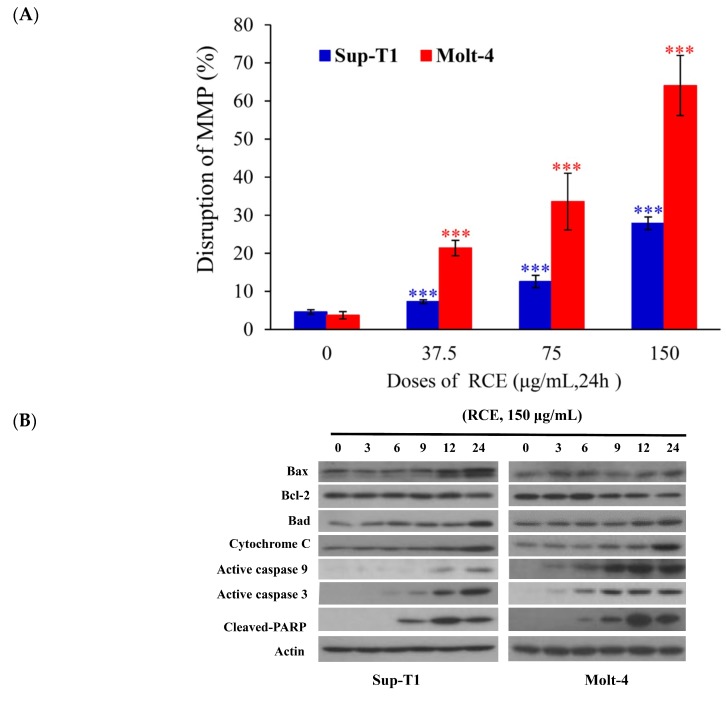

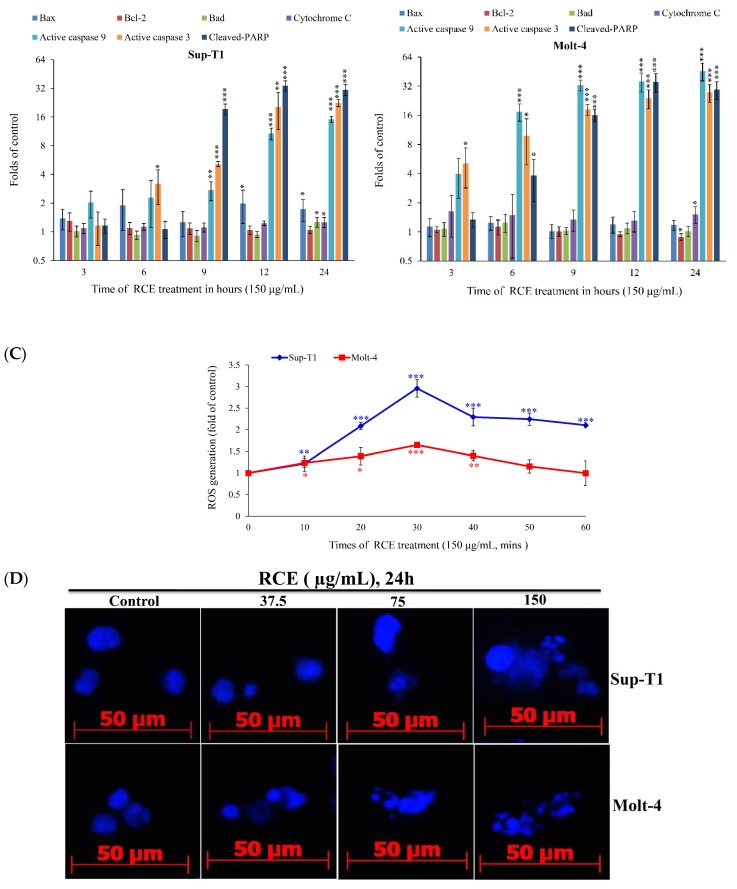
RCE treatment induces mitochondrial membrane potential (MMP) disruption and redox oxygen species (ROS) production in Sup-T1 and Molt-4 cells. Cells were treated with the indicated concentrations of RCE for 24 h; (**A**) Mitochondrial membrane potential of Sup-T1 and Molt-4 cells were assessed with JC-1 staining using flow cytometric analysis; (**B**) The effect of RCE on the expressions of mitochondria-related proteins of Sup-T1 and Molt-4 cells was examined with Western blotting assay. Actin was used as the loading control. The target protein was compared with the respective actin and then compared with the control group. The blots were quantified using the Odyssey image analysis system with National Institutes of Health ImageJ software. Cells were treated with RCE (150 μg/mL) for 3, 6, 9, 12, and 24 h; (**C**) Cells were treated with RCE (150 μg/mL) for the indicated times. Quantitative results showed a gradual increase in ROS production in response to RCE treatment when compared with the control group. The results are presented as mean ± SD of three independent experiments (* *p* < 0.05; ** *p* < 0.01; *** *p* < 0.001); (**D**) The changes of the nuclear morphology were determined with DAPI staining and observed in Sup-T1 and Molt-4 cells using a fluorescent microscope (400×). All results are expressed as the mean ± standard deviation of three independent experiments. *p*-values of statistical significance are represented as * *p* < 0.05, ** *p* < 0.01, and *** *p* < 0.001.

Previous studies demonstrated that the cytotoxic effect of many anticancer drugs involved the induction of oxidative radicals and mitochondrial dysfunction [30,31]. In the current study, RCE induced Molt-4 and Sup-T1 cell apoptosis, evidenced by annexin-V/PI staining (Figure 1C). To fully understand the apoptotic effect of RCE, we investigated whether the induction of apoptosis involved the interruption of mitochondrial membrane potential (MMP) and this was achieved using JC-1 dye staining. RCE (37.5 μg/mL) increased the population of Molt-4 and Sup-T1 cells with disrupted MMP to 7.28% and 21.38%, respectively (Figure 3A). The disruption of MMP was significantly intensified with the use of higher doses of RCE. The use of RCE (75 and 150 μg/mL) increased the population of Sup-T1 cells with disrupted MMP to 12.56% and 27.86% as well as Molt-4 cells reaching 33.56% and 64.06%, respectively. To examine the effect of RCE treatment on the induction of the intrinsic pathway, Western blotting was performed in RCE-treated Sup-T1 and Molt-4 cells. RCE increased the expression of Bax, Bad, and cytochrome *c* as well as the cleavage of caspases-3, -9, and PARP (Cleavage of PARP was considered as a hallmark of apoptosis [32,33]), but attenuated the expression of Bcl-2 (Figure 3B).

To understand the full picture of RCE cytotoxic activity, we examined the effect of RCE on the induction of oxidative stress. Cells were treated with RCE and the levels of ROS at different time intervals were determined. ROS generation was increased in a time-dependent manner and monitored using carboxy-H_2_DCFDA dye, a carboxy derivative of fluorescein dye. RCE (150 μg/mL) administration for 10, 20, 30, 40, 50, and 60 min resulted in 1.21-, 2.08-, 2.96-, 2.29-, 2.25-, and 2.1-fold and 1.23-, 1.38-, 1.64-, 1.39-, 1.15- and 0.99-fold increase in the ROS levels in Sup-T1 and Molt-4 cells, respectively (Figure 3C). DAPI staining with a fluorescent microscope was used to assess the nuclear morphological changes induced by RCE in Sup-T1 and Molt-4 cells. The number of condensed nuclei in RCE-treated cells was much higher than the control, which showed intact and normal nuclei (Figure 3D). These findings indicated that the apoptotic effect of RCE involved oxidative stress.

### 2.3. Effect of RCE on ER Stress and DNA Damage

**Figure 4:**
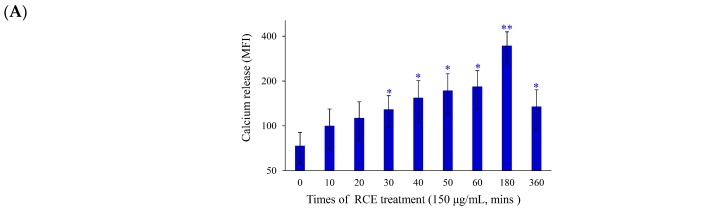

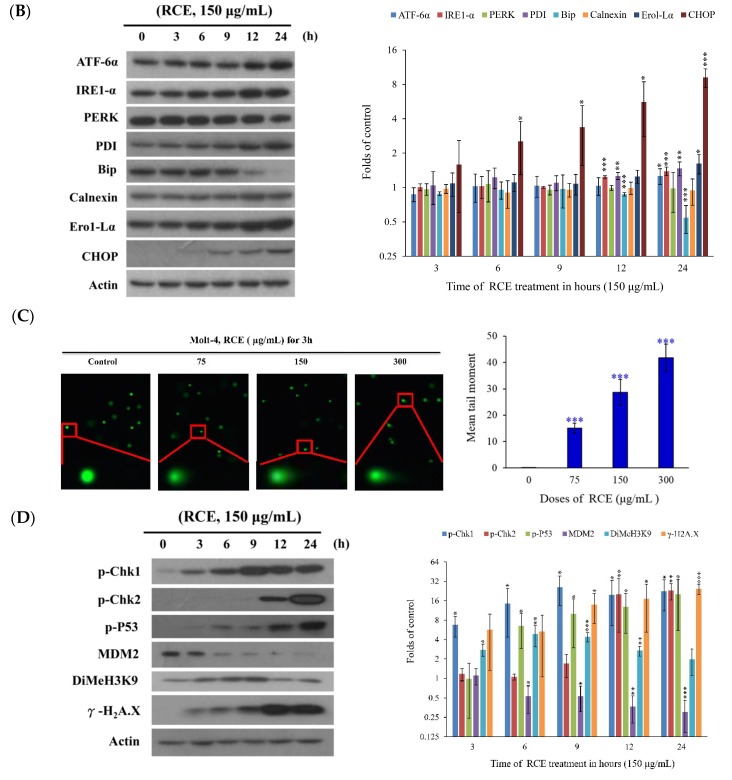
Effect of RCE on the induction of ER stress and DNA damage in Molt-4 cells. (**A**) Cells were treated with RCE (150 μg/mL) for the indicated times. Quantitative results showed a gradual increase in calcium accumulation in response to RCE treatment when compared with the control group; (**B**) The effect of RCE on the expression of ER stress-related proteins of Molt-4 cells was examined with Western blotting assay. The target protein was compared with the respective actin and then compared with the control group. The blots were quantified using the Odyssey image analysis system with ImageJ software. Cells were treated with 150 μg/mL RCE for the indicated times; (**C**) An example of the “comet tail” due to chromosomal DNA double-strand breaks in RCE-treated Molt-4 cells (0, 37.5, 75, and 150 µg/mL) compared with the untreated control. Electrophoresis was carried out under neutral conditions. Quantitative results showed a gradual increase in the tail movement upon RCE treatment when compared with the control. Results are presented as mean ± SD of three independent experiments (*** *p* < 0.001) (400×); (**D**) Cells were harvested, and the lysates were prepared and subjected to SDS-PAGE followed by immunoblotting of DNA damage-related proteins. Actin was used as the loading control in B and D.

As previously demonstrated, the induction of endoplasmic reticulum (ER) stress and DNA damage played a major role in the cytotoxic effect of certain anticancer agents [30]. The effect of RCE on the induction of ER stress in Molt-4 cells was investigated. In addition, we examined the calcium concentration in Molt-4 cells with staining of Fluo-3, a fluorescence indicator of intracellular calcium, after treatment with RCE to determine if its apoptotic effect involves the interruption of calcium homeostasis. Ca^2+^ concentration increased 1.34-, 1.52-, 1.75-, 2.07-, 2.32-, 2.47-, 3.43-, and 1.33-fold after RCE treatment (150 µg/mL) for 10, 20, 30, 40, 50, 60, 180, and 360 min, respectively, as compared with the solvent control (Figure 4A). We were interested in studying the effect of RCE on ER and calcium hemostasis because recent reports indicated that ER stress-related processes including the unfolded protein response (UPR) and calcium homeostasis represented attractive anticancer targets [34]. RCE treatment disrupted calcium homeostasis as demonstrated by our results (Figure 4A). Previous studies indicated that the depletion of calcium from ER disturbs ER chaperone functions and triggers ER stress, resulting in the activation of the unfolded protein responses [34,35]. To understand if RCE effect on Molt-4 cells involves these targets, three major transmembrane transducer proteins including protein kinase RNA-like ER kinase (PERK), activating transcription factor 6α(ATF-6α), and inositol-requiring protein-1α (IRE-1α), as well as ER chaperones (protein disulfide isomerase (PDI), Bip, calnexin, endoplasmic reticulum oxidoreductin 1-Lα (Ero1-Lα), and C/EBP homologous protein (CHOP), were determined using Western blotting. In a time-dependent manner, RCE treatment suppressed the expressions of PERK and Bip, whereas the expressions of ATF-6α, IRE-1α, PDI, calnexin, ERO-Lα and CHOP were significantly increased (Figure 4B).

To examine whether the effect of RCE on DNA damage could be related to apoptosis induction, comet and Western blotting assays were performed. In a dose-dependent manner, RCE (75, 150, and 300 µg/mL) promoted DNA migration in Molt-4 cells which was demonstrated by the increase of DNA fragmentation (Figure 4C). RCE treatment led to the activation of cell cycle checkpoints in Molt-4 cells. This effect was evaluated by checking RCE effect on the phosphorylation of checkpoint kinases 1 and 2 (chk1 and 2), and H2A histone family member X (H2A.X) as well as the expression of murine double minute 2 (MDM2), and dimethylation of histone H3 lysine 9 (DiMeH3K9). Treatment with 150 µg/mL of RCE initially accelerated the phosphorylation of Chk1 at serine 345 and the dimethylation of histone H3 lysine 9 after 3 h. Then, the phosphorylation of H2A.X at serine 139 (γ-H2A.X) and p53 at serine 15 was promoted after 9 h. In contrast, the expression of MDM2 was attenuated 6 h after treatment (Figure 4D). Our results indicated that the antitumor effect of RCE was strongly associated with the induction of ER stress and nuclear DNA damage in Molt-4 cells.

### 2.4. Apoptotic Effect of RCE Involves the Dysregulation of Nrf2/Keap1/p62 Pathway

**Figure 5:**
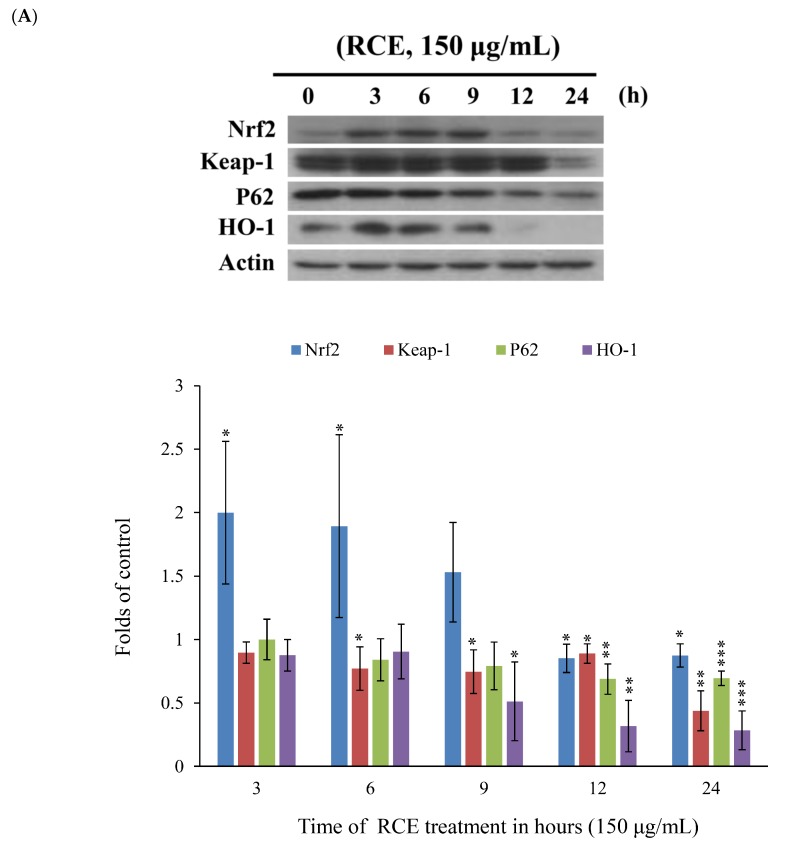

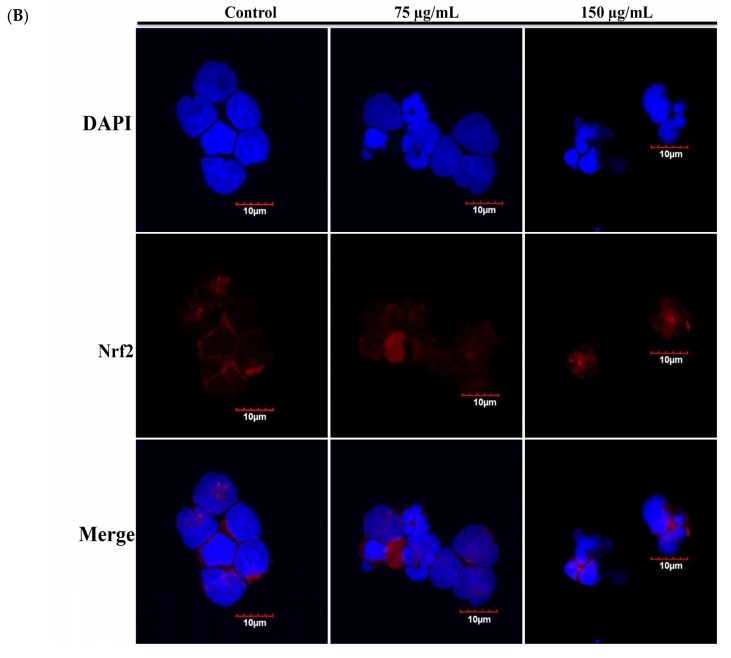

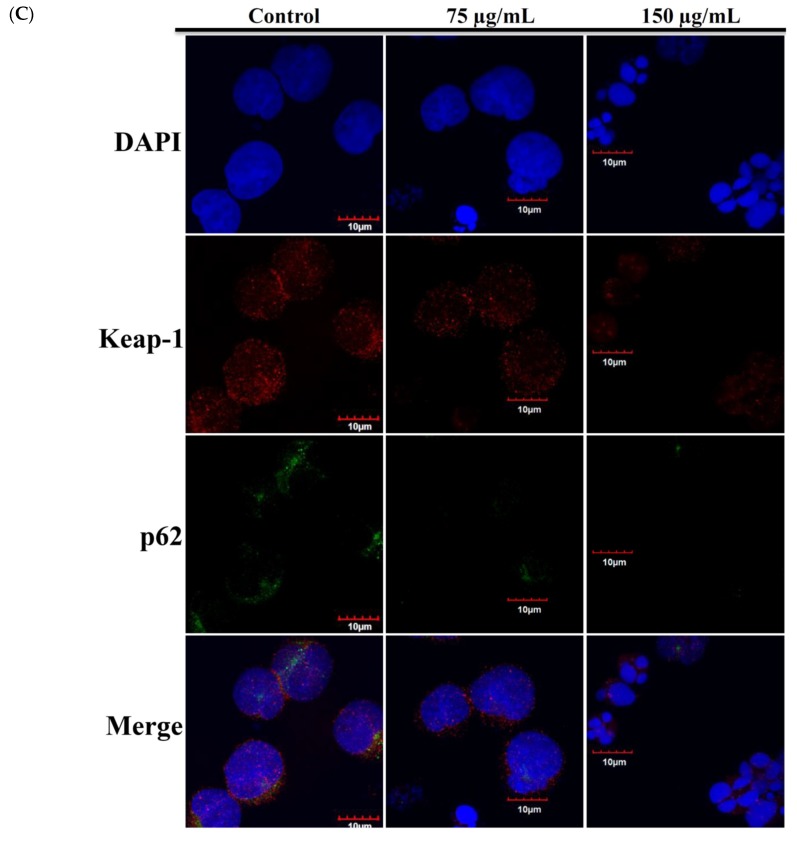
Effect of RCE on Nrf2/Keap1/p62 pathway. (**A**) Effect of RCE on the expressions of Nrf2/Keap1/p62 proteins. Cells were harvested, and the lysates were prepared and subjected to SDS-PAGE followed by immunoblotting of Nrf2/Keap1/p62-related proteins. Actin was used as the loading control. The target protein was compared with the respective actin and then compared with the control group. The blots were quantified using the Odyssey image analysis system with ImageJ software; (**B**,**C**) Effect of RCE on the expressions of Nrf2/Keap1/p62 proteins with the immunofluorescent method using confocal analysis. Cells were treated with 75 and 150 µg/mL of RCE for 24 h.

Recent studies indicated that nuclear factor erythroid 2-related factor2 (Nrf2)/Kelch-like ECH-associated protein 1 (keap1) pathway participated in the protection of cells from oxidative stress, chemotherapeutic agents, and radiotherapy [36,37,38,39]. It was suggested that Nrf2 transcription factor participates in reducing the effect of H_2_O_2_ by regulating the inducible expression of numerous antioxidant and detoxification enzymes [40,41]. To study the expression of Nrf2/Keap1/p62 protein cascades in Molt-4 cells and to demonstrate that RCE effectively blocked the antioxidative signaling pathway, we sought to evaluate the expression of Nrf2/Keap1/p62 pathway following the oxidative stress-induced by RCE. Results of Western blotting showed a minor and transient increase in Nrf2 expression. However, the expressions of p62 and heme oxygenase-1 (HO-1) were significantly attenuated with RCE treatment in a time-dependent manner (Figure 5A). The immunofluorescent method was used to detect the localization of Nrf2, Keap1, and p62 proteins. The use of RCE (75 and 150 μg/mL) for 24 h significantly attenuated the accumulation of Nrf 2, p62, and Keap1 of Molt-4 cells, in agreement with the results of Western blotting (Figure 5B,C).

### 2.5. The Antitumor Effect of RCE Is Associated with Overexpression of PTEN Protein

Since several signaling cascades, MAPKs, PI3K/Akt and Jak/Stat, are involved in the functions of Nrf2 [42,43,44,45], we therefore examined the involvement of these signaling pathways in cellular response to RCE treatment. Cells were treated with RCE for 3, 6, 9, and 18 h, and the expressions of these cascades’ proteins were evaluated by Western blotting. RCE treatment resulted in the promotion of p-PTEN, p-c-Raf, and phosphorylation of extracellular signal-regulated kinase (p-ERK). However, the phosphorylations of other proteins were decreased in a time-dependent manner (Figures S4 and S5).

**Figure 6:**
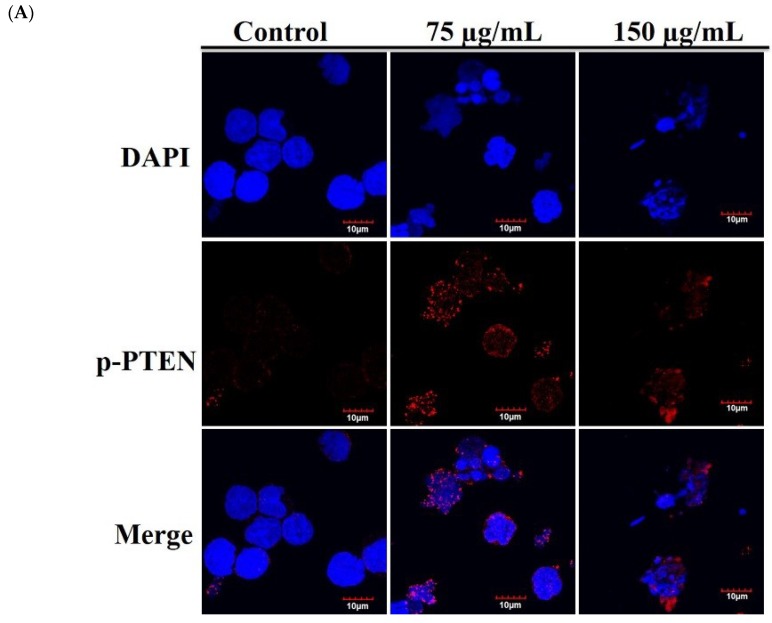

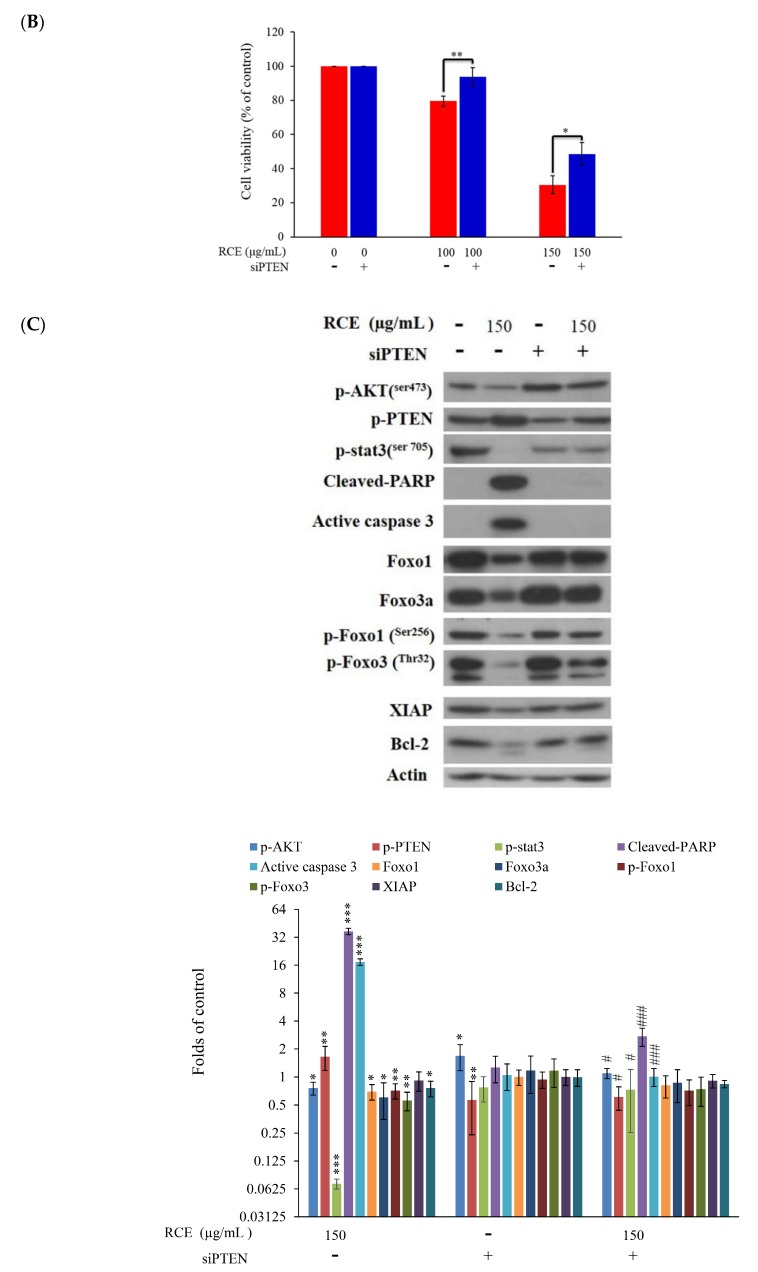
Antitumor effect of RCE involves the disruption of MAPK, PI3K/Akt and Jak/Stat signaling pathways. (**A**) Effect of RCE on the localization of p-PTEN protein (1000×) and the cells were treated with 75 and 150 µg/mL of RCE for 24 h; (**B**,**C**) The expression of phosphatase and tensin homolog (PTEN) was knocked down by RNAi and the cells were then treated with 150 µg/mL of RCE for 48 h. Cell growth and proteins’ expressions were determined with MTT and Western blotting assays, respectively. Actin was used as the loading control. The target protein was compared with the respective actin and then compared with the control group. The blots were quantified using the Odyssey image analysis system with ImageJ software. * *p* < 0.05, ** *p* < 0.01, and *** *p* < 0.001 as compared with the control; # *p* < 0.05 and ### *p* < 0.001 as compared with RCE treatment group.

T-cell leukemogenesis is dependent on the mutation of PTEN inactivation, which leads to aberrant activation of oncogenic transcription factors in leukemia patients [20]. Cytoplasmic PTEN is supposed to repress apoptosis and enhance cell growth, whereas nucleus PTEN regulates cell cycle progression by controlling p70^S6K^ [46]. We used immunofluorescence assay by confocal microscope to examine the localization of p-PTEN after RCE treatment. The use of RCE resulted in the punctate formation of p-PTEN compared with the untreated Molt-4 cells (Figure 6A). In agreement with our results, it was reported that the accumulation of p-PTEN in the nucleus resulted in the suppression of PI3K/AKT/GSK and Jak/Stat signaling pathways [47]. To confirm if RCE had similar activity against HEK 293 cells, we examined the effect of RCE on mitochondrial integrity, homeostasis of ROS, and calcium. RCE displayed a similar induction of MMP disruption as well as ROS and ER stress in HEK 293 cells (Figure S6A–C, Supplementary Materials). PTEN plays an important role in apoptosis and finally, we further examined the role of PTEN activation in the apoptotic effect of RCE on HEK 293 cells by RNAi technology. As shown in Figure 6B, knocking down PTEN phosphorylation suppressed the growth inhibitory effect of RCE (100 and 150 µg/mL) on HEK 293 cells to about 14.1% and 18.1%, respectively. The level of PTEN phosphorylation in siRNA-treated HEK 293 cells was reduced as revealed by immunoblotting analysis. The expressions of downstream target genes, Akt, Stat3, and Foxo, as well as survival proteins XIAP and Bcl-2, were attenuated by RCE treatment but restored in PTEN-knocked down cells. In addition, the cleavage of PARP and caspase-3 was enhanced by RCE treatment but was significantly eliminated in PTEN-knocked down cells (Figure 6C). These results indicated that PTEN-knocked down HEK 293 cells reversed the apoptotic effect of RCE.

## 3. Discussion

The treatment protocol of adult ALL patients usually consists of a standard high dose of chemotherapy. However, the outcomes are still unfavorable, and usually poor in elderly patients. Survival rates sharply declined with the emergence of chemoresistance resulting in higher mortality rates in ALL patients. Although there was marked progress in the treatment of B-ALL patients, the development of new agents for T-ALL patients still lagged. Thus, it is crucial to find more effective antileukemic compounds with better safety profile. Mother Nature has been and will continue to be the treasure trove for biologically active compounds. Our study showed that RCE, ethanolic extract of *R. cymosa* fruits, inhibited tumor growth in both in vitro and in vivo models by inducing apoptosis. When lymphoblastic lymphoma Sup-T1 cells and leukemia Molt-4 cells were exposed to RCE at 150 μg/mL for 24 h, their proliferation percentages were suppressed to 35.38% and 24.38%, respectively (Figure 1B). RCE treatment of Sup-T1 and Molt-4 cells induced DNA damage, ROS (increased within 30 min then decreased) generation, ER stress, and mitochondrial dysfunction resulting in apoptotic cell death. Apoptosis induction in cancer cells is one of the important goals of chemotherapy. Previous studies suggested that the increase in ROS levels can be modulated by various redox enzymes including oxidoreductases [48]. Similar transient increase in ROS levels was noticed in response to certain cytotoxic compounds including sodium selenite and resveratrol [49,50].

Our results suggested that Molt-4 cells were more susceptible to RCE treatment than SupT1 cells, so we selected Molt-4 cells to reveal the RCE cytotoxic mechanism of action. In addition, we were interested in evaluating the antitumor effect of *R. cymosa* in the animal model to provide a full picture of its potential application as an antileukemic agent. Antileukemic activity of RCE and RCP was assessed by evaluating the tumor growth of human lymphoblastic leukemia Molt-4 cells in the xenograft animal model. Our study indicated that the administration of RCE and RCP significantly suppressed the growth of Molt-4 tumors compared with the solvent control.

It was reported that Nrf2 inactivation is a result of the suppression of protein–protein interaction between phosphorylated p62 and Keap1 [18]. Keap1 acts as a cytoplasmic repressor protein that tightly regulates the degradation of Nrf2 located in the cytoplasm [15,19]. Nrf2 is released from the Keap1 complex and is translocated to the nucleus to induce phase II cytoprotective genes by binding to the antioxidant-related elements (AREs) under electrophilic and oxidative stress [42]. The expression of ARE-driven HO-1 gene is controlled by Nrf2, a key transcription factor [51]. Recent data have shown that the physical and functional interaction of p62, which is a binding partner of Keap1, along with Keap1 regulates the Keap1-Nrf2 cell defense pathway and aids in its degradation [20]. The accumulation of Keap1 interacting proteins, p62 and p21, blocks Nrf2 binding to Keap1, leading to Nrf2 accumulation [52,53]. Therefore, Keap1-bound Nrf2 is released and translocated inside the nucleus of cells [39]. We found that the expressions of p62 and HO-1 proteins were attenuated with RCE treatment in a time-dependent manner and the initial overexpressions of Nrf2/Keap1 were completely suppressed after 24 h, as demonstrated by Western blotting and confocal analysis (Figure 5). Recent studies have demonstrated that the regulation of Keap1-Nrf2 is an important therapeutic target for tumorigenesis and neurodegenerative disorders [39,54]. Therefore, the regulatory role of Nrf2/Keap1/p62 signaling pathways should be fully elaborated for RCE to be developed as a cytotoxic remedy.

A deeper knowledge of tumorigenesis should help us to develop new chemotherapeutic agents to attack cancer. The new insights on the biology and heterogeneity of leukemogenesis not only revealed a novel function for oncogenes but also provided an alternative therapy for a previously unconsidered model of leukemia. Recent results have demonstrated that the losses or aberrations of PTEN were significantly and frequently observed in several human cancers [20,55,56,57]. It was also found that the de novo acquired PTEN inactivation in Notch1-dependent leukemia could temporarily activate the PI3K/Akt signaling pathway resulting in an increase in glycolysis [20]. FOXOs transcription factors are downstream effectors of AKT that scavenge ROS by regulating the expression of detoxifying enzymes in oxidative stress [58]. AKT directly phosphorylates FOXOs and thus excludes these factors from the nucleus [59]. HO-1 regulates intracellular ROS by inducing antioxidant enzymes and thus can mediate the inhibition of cell apoptosis [60]. The activation of Jak/Stat and NFκB signaling pathways regulates cancer development and progression [61]. Following the treatment of Molt-4 cells with RCE for the indicated time intervals, a significant suppression in the expressions of PI3K/Akt/Foxo/HO-1 and Jak/Stat signaling cascades was observed. In the present study, we observed that the phosphorylation of PTEN and ERK was gradually elicited in a time-dependent manner (Figure 6). Surprisingly, siPTEN effectively attenuated the inhibition of cell growth and activation of Akt, Foxo, Stat 3, caspases -3 and -9 as well as the cleavage of PARP (Figure 6). PTEN is a major lipid phosphatase that prevents Akt activation and thus induces the activation of specific resistant genes (CREB and MDM-2) or inactivation of proapoptotic proteins (caspases and p21) [62]. Therefore, it was suggested that the cytotoxic effect of RCE was partially promoted through the activation of PTEN.

Our findings demonstrated that the ethanolic extract of *R. cymosa* fruits (RCE) exhibited antileukemia effect through ROS- and ER stress-mediated apoptosis leading to Molt-4 cell death via the overexpression of PTEN and the dysregulation of PI3K/Akt and Jak/Stat 3 signaling pathways. In addition, we identified PTEN as the molecular target of RCE. Our data suggested that the loss of PTEN expression not only attenuated cell growth but also suppressed the cleavage of PARP and promoted the phosphorylation of Akt and Jak/Stat 3 as well as the expressions of XIAP and Bcl-2 in Molt-4-treated RCE. These results indicated that the activation of PTEN, a tumor suppressor gene, might contribute partially to the antitumor effect of RCE. PTEN negatively regulates the PI3K/AKT signaling pathway and is often inactivated in T-ALL patients. Thus, the aberrations in PTEN were identified as a significant and independent risk factor for relapse-free survival and overall survival in T-ALL patients treated with different chemotherapeutic protocols. Furthermore, a novel therapeutic target, NOCTH1 inhibition, was found to be associated with PTEN-AKT signaling pathway [20,63]. The restoration of PTEN function may be a crucial step in achieving better survival rates and preventing disease relapse with patients receiving chemotherapy [64,65].

## 4. Materials and Methods

### 4.1. Chemicals and Biological Materials

Cell lines were obtained from American Type Culture Collection (ATCC, Manassas, VA, USA). the cells were kept at 37 °C in 5% CO_2_ (humidified atmosphere). A growing medium of RPMI 1640 medium was used, which was supplemented with glutamine (2 mM), antibiotics (100 μg/mL of streptomycin and 100 units/mL of penicillin), and fetal calf serum (10%). Antibodies against caspases-3 and 9, cleaved PARP, Foxo1, Foxo3a, p-Foxo1 (Ser256), p-Foxo 3 (Thr32), p-Chk1, p-Chk2, MDM2, DiMeh3k9, Nrf2, Keap1, P62, HO-1, PDI, Bip, Calnexin, Ero1-Lα, and p-Stat 3 (Tyr705) were obtained from Cell Signaling Technologies (Beverly, MA, USA). Streptomycin, fetal calf serum (FCS), trypan blue, RPMI 1640 medium, and penicillin G were purchased from GibcoBRL (Gaithersburg, MD, USA). Antibodies for Bad, Bax, Bcl-2, cytochrome *c*, p-Akt (Ser473), p-PTEN, XIAP, IRE1-α, PERK, p-P53, γ-H_2_A.X, ATF-6α, and CHOP were purchased from Santa Cruz Biotechnology (Santa Cruz, CA, USA). Anti-mouse and rabbit IgG peroxidase-conjugated secondary antibodies were purchased from Pierce (Rockford, IL, USA). Annexin V-FITC/PI (propidium iodide) stain was obtained from Strong Biotech Corporation (Taipei, Taiwan). 3-(4,5-Dimethylthiazol-2-yl)-2,5-diphenyl-tetrazolium bromide (MTT) and dimethyl sulfoxide (DMSO), and all other chemicals were obtained from Sigma-Aldrich (St. Louis, MO, USA). Carboxy derivative of fluorescein (carboxy-H_2_DCFDA), Fluo-3, and JC-1 cationic dye were obtained from Molecular Probes and Invitrogen technologies (Carlsbad, CA, USA). ECL Western blotting detection kits and Hybond ECL transfer membrane were obtained from Amersham Life Sciences (Amersham, UK).

### 4.2. Preparation of Ethanol Extract from R. cymosa (RCE)

*R. cymosa* fruits were identified by Dr. Tzen-Yuh Chiang at the Department of Life Sciences, National Cheng Kung University (Figure S1, Supplementary Materials). Then, 700 g of the dried fruits were ground into fine powder and refluxed with ethanol in a 1:10 (*w*/*v*) ratio. The extract was kept in the dark and left to precipitate overnight at room temperature. The extract supernatant was filtered by a filter paper, centrifuged at 3000 rpm for 30 min to remove the precipitate, and then the extract was lyophilized and stored at −70 °C. RCE was dissolved in DMSO and was further used in the in vitro experiments.

### 4.3. Anti-Oxidative Activity Assay

The free radical scavenging activity was performed using DPPH (1,1-diphenyl-2-picrylhydrazyl) assay. Samples (120 mL) were diluted to the indicated concentrations and mixed with 80 mL of DPPH (1 mM) for 30 min and the absorbance was measured at 550 nm.

### 4.4. MTT Cell Proliferation Assay

Culture plates (96-well) were used in the MTT assay. The cells were seeded at 4 × 10^4^ per well and then treated with the tested materials at several concentrations [66,67]. The cytotoxic effect of the extract was examined by MTT assay (thiazolyl blue tetrazolium bromide, Sigma-M2128) for 24, 48, or 72 h. An ELISA reader (Anthoslabtec Instrument, Salzburg, Austria) was used to measure the absorbance values, at 570 and 620 nm (OD = OD_570_ − OD_620_). The concentration that caused 50% inhibition (IC_50_) was calculated using CalcuSyn software (Version 1.1.1, Biosoft, Ferguson, MO, USA). The results are presented as mean ± SD of three independent experiments.

### 4.5. Annexin V/PI Apoptotic Assay

Annexin V-FITC staining kit was used to determine membrane integrity and phosphatidylserine (PS) externalization [66]. Cells (10^6^) were grown in 35 mm diameter plates, annexin V-FITC (10 μg/mL) and PI (20 μg/mL) were used to label the cells. A binding buffer was used to wash all plates and then the cells were harvested. A binding buffer was used to suspend the cells (2 × 10^5^ cells/mL) and the label was assessed using a FACS-Caliburflow cytometer (Beckman Coulter, Taipei, Taiwan). The results were analyzed with CellQuest software (BD CellQuest Pro, Franklin Lakes, NJ, USA). Approximately 10,000 cells were counted for each measurement.

### 4.6. Determination of ROS Generation, MMP Disruption, and Calcium Accumulation

Previous reports have described how to determine calcium accumulation, MMP disruption, and ROS generation [66]. Calcium accumulation, MMP disruption, and ROS generation were evaluated with fluorescent calcium indicator (Fluo 3, 5 mM), JC-1 cationic dye (1.25 μg/mL), and the carboxy derivative of fluorescein (carboxy-H_2_DCFDA, 1.0 mM), respectively. Cells were labeled with a specific fluorescent dye for 30 min after treating the cells with the tested extract. PBS was used to wash and resuspend cells (1 × 10^6^ cells/mL) after labeling. Flow cytometry was used to investigate stained cells.

### 4.7. Western Blot Analysis

Cells were treated for 30 min with RIPA lysis buffer (1% Nonidet P-40, 0.1% sodium dodecyl sulfate (SDS), 1× PBS, 1 mM sodium orthovanadate, 0.5% sodium deoxycholate, 100 μg/mL phenylmethylsulfonyl Xuoride, and 30 μg/mL aprotinin) (all chemicals were obtained from Sigma-Aldrich) to obtain cell lysates [67]. The lysates were centrifuged at 20,000× *g* for 30 min and the protein concentration in the supernatant was determined using a BCA protein assay kit (Pierce, Rockford, IL, USA). SDS-polyacrylamide gel electrophoresis (7.5%, 10%, or 12%) was used to separate equal amounts of proteins which were then electrotransferred to a PVDF membrane (PVDF membranes had a high binding affinity for proteins and used for application western blotting). A solution containing 5% nonfat dried milk TBST buffer (20 mM Tris-HCl, pH 7.4, 150 mM NaCl, and 0.1% Tween 20) was used to block the membrane for 1 h, which was then washed with TBST buffer. Using an immunoblotting assay with specific antibodies, the protein expressions were monitored. An enhanced chemiluminescence kit (Pierce, Rockford, IL, USA) was used to detect these proteins.

### 4.8. Immunofluorescence Analysis

The tested extract was used to treat cells which were then fixed with 4% paraformaldehyde in 50 mM HEPES buffer (pH 7.3) for 30 min. To permeabilize cells, Triton X-100 (0.2%) in PBS (pH 7.4) was used for 20 min. Cells were incubated with 5% BSA (BSA was a globular protein that was used in numerous biochemical applications due to it stability in biological reactions) in PBS containing 0.05% Triton X-100 (T-PBS) for 1 h. To avoid non-specific protein binding, the experiment was done at room temperature. Cells were incubated with the primary antibodies (1:1000) for 2 h. Secondary antibodies (Alexa Fluor 586-conjugated goat anti-mouse IgG (H+L) (Life Technologies, Carlsbad, CA, USA) were diluted at 1:1000 for 1 h at room temperature. DAPI (1 μg/mL) was used as a counterstain for the nuclei. PBS was used to wash the cells, which were then observed with a FV1000 confocal laser scanning microscope (Olympus, Tokyo, Japan).

### 4.9. RNA Interference Transfection

In a six-well plate, HEK 293 cells were plated and transfected with 20 nM of ON-TARGET plus human PTEN (5728) siRNA (GE Healthcare Dharmacon, Lafayette, CO, USA) using Lipofectamine^TM^ 2000 transfection reagent (Invitrogen, Carlsbad, CA, USA) according to the manufacturer’s protocol in the serum-free Opti-MEM medium. After 6 h, the fresh medium with 10% FBS was replaced, and the experiments were performed after 48 h of transfection.

### 4.10. Xenograft Animal Model with Human Leukemia Molt-4 Cells

The xenograft animal model was established in nude mice as reported in a previous study [23]. The source of six-week-old male immunodeficient athymic mice was the National Laboratory Animal and Research Center (Taipei, Taiwan). Standard laboratory conditions (temperature 24–26 °C, 12–12 h dark-light circle) were maintained for the mice. A laboratory diet and water were used to feed the mice. The recommendations in the guidelines of the Guide for the Care and Use of Laboratory Animals of the National Institutes of Health were strictly followed. All efforts were implemented to minimize animal stress/distress. In 0.2 mL PBS, Molt-4 cells (1 × 10^6^) were suspended and then injected into the right flank of each mouse subcutaneously. Every day the tumor growth was monitored. After fourteen days, mice with confirmed tumor growth were randomly divided into three groups. The treatment group received RCE (50 µg/g) or RCP (250 µg/g). The control group received only the solvent (PBS). All treatments were administered orally. RCE and RCP were administered on every other day for 60 days. Mice were sacrificed by carbon dioxide. The tumor volume was measured using calipers three times a week. The following equation, width^2^ × length/2, was used to measure the tumor volume.

### 4.11. Neutral Comet Assay for the Detection of DNA Double-Strand Breaks (DSBs)

The assay was carried out using a CometAssay^TM^ Kit (Trevigen, Gaithersburg, MD, USA) following the manufacturer’s protocol for the neutral comet assay. Briefly, cancer cells (2 × 10^5^ cells/mL) were treated with RCE at the indicated concentrations. Cells were combined with 1% low melting point agarose at a ratio of 1:10 (*v*/*v*) and immediately a 75 μL portion of the mixture was pipetted onto CometSlide^TM^ and the slides were immersed in ice-cold lysis solution (Trevigen) for 30 to 60 min. The slides were placed in a horizontal electrophoresis apparatus and electrophoresed in 1× TBE (90 mM Tris-HCl, 90 mM boric acid, and 2 mM EDTA, pH 8.0) at 20 V for 10 min. The samples were then fixed in 70% ethanol and dried before stained with 1:10,000 SYBR Green I (Trevigen) to visualize cellular DNA. In order to quantify DNA damage, the fluorescence images were analyzed using the TriTek Comet Image according to the length of the comet tail and the integrated fluorescence values of each defined area were recorded. The comet length was measured from the trailing edge of the nucleus to the leading edge of the tail. Calculations were averaged per replicate.

### 4.12. Statistics

The results were expressed as the mean ± standard deviation (SD). An unpaired Student’s *t*-test was used to compare each experiment. A *p*-value of less than 0.05 was statistically significant.

## 5. Conclusions

The treatment of lymphoblastic lymphoma Sut-T1 cells and leukemia Molt-4 cells with RCE induced oxidative stress, ER stress, and mitochondrial dysfunction resulting in apoptosis. These effects were detected with annexin V/PI, JC-1, Fluo-3, and carboxy-H_2_DCFDA assays. It was confirmed that RCE treatment suppressed protein expressions of various signaling pathways involved in tumorigenesis. Our findings suggested that the tumor suppressor gene, PTEN, contributed to the apoptotic effect of RCE on leukemia cells. Accumulating evidence suggested that the majority of prevalent pathogenic mechanisms, PTEN/PI3K/Akt, could provide a compelling promise for the treatment of hematologic cancers. Our study shed light on the antitumor mechanism of RCE and its potential applications as a novel PTEN inducer to treat patients with T-cell lymphoblastic leukemia.

## Supplementary Materials

Supplementary materials can be found at https://www.mdpi.com/1422-0067/20/8/1935/s1.

## Abbreviations

RCE	Ethanolic extract of *R. cymosa* fruits
RCP	Fruit powder of *R. cymosa* fruits
MMP	Mitochondrial membrane potential
PTEN	Phosphatase and tensin homolog
ALL	Acute lymphoblastic leukemia
ROS	Redox oxygen species
AREs	Antioxidant-related elements
PERK	Protein kinase RNA-like ER kinase
ATF-6α	Activating transcription factor 6α
IRE-1α	Inositol-requiring protein-1α
PDI	Protein disulfide isomerase
Ero1-Lα	Endoplasmic reticulum oxidoreductin 1-Lα
CHOP	C/EBP homologous protein
Nrf2	Nuclear factor erythroid 2-related factor2
keap1	Kelch-like ECH-associated protein 1
HO-1	Heme oxygenase-1
