# Supplementary material for: The Antioxidant from Ethanolic Extract of Rosa cymosa Fruits Activates Phosphatase and Tensin Homolog In Vitro and In Vivo: A New Insight on Its Antileukemic Effect
Source: Int J Mol Sci. 2019 Apr 19;20(8):1935. doi: 10.3390/ijms20081935 (PMC6514837; doi:10.3390/ijms20081935)
Supplement: Supplementary file 1 [file ijms-20-01935-s001.pdf]

Supplementary Files

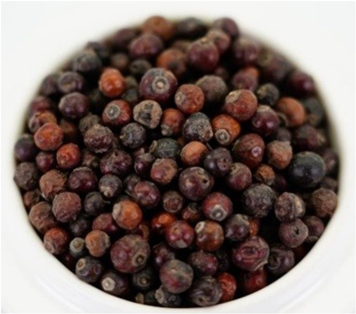

Figure S1. The morphology of *Rosa cymosa* fruits.

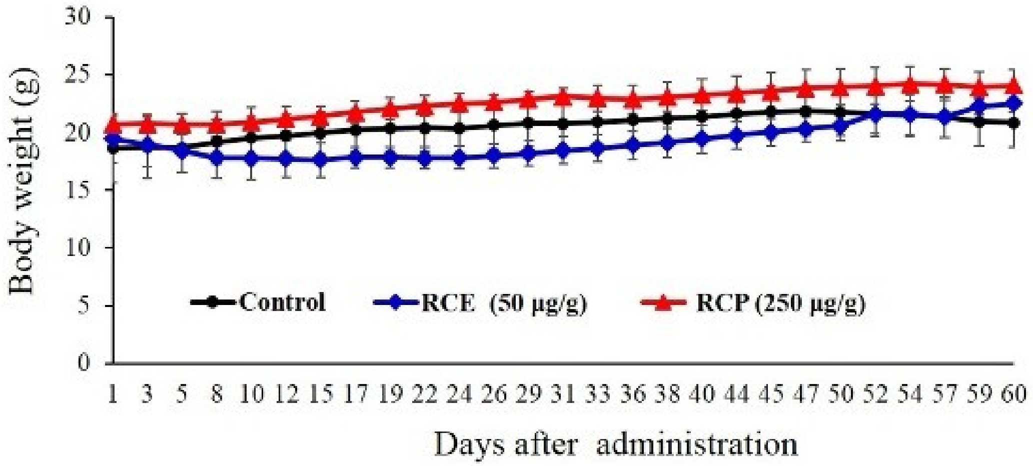

Figure S2. Mice body weights were measured three times per week.

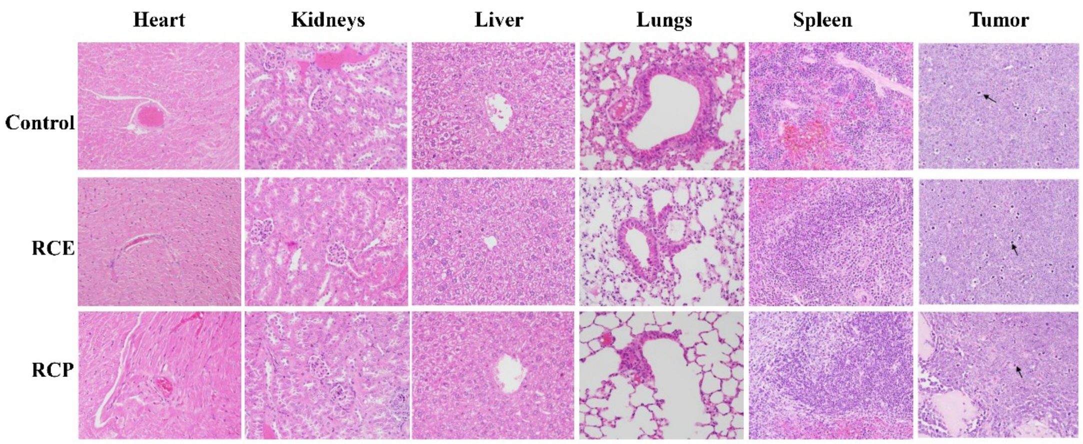

Figure S3. Tissue sections from mouse organs were stained with hematoxylin and eosin

using an optical microscope (400×).Tumor cells expressed single oval round, high nucleic/cytoplasm ratio with highly mitosis (arrow).

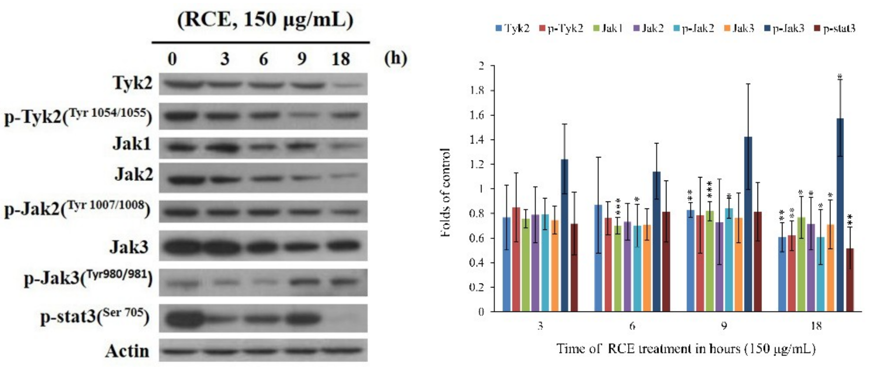

Figure S4. Effect of RCE on the expression of Tyk2/Jak signaling pathway. Actin was used as the loading control. The target protein was compared with the respective actin then compared with the control group. The blots were quantified using the Odyssey image analysis system with ImageJ software.*$p < 0.05$, **$p < 0.01$, and ***$p < 0.001$ as compared with the control

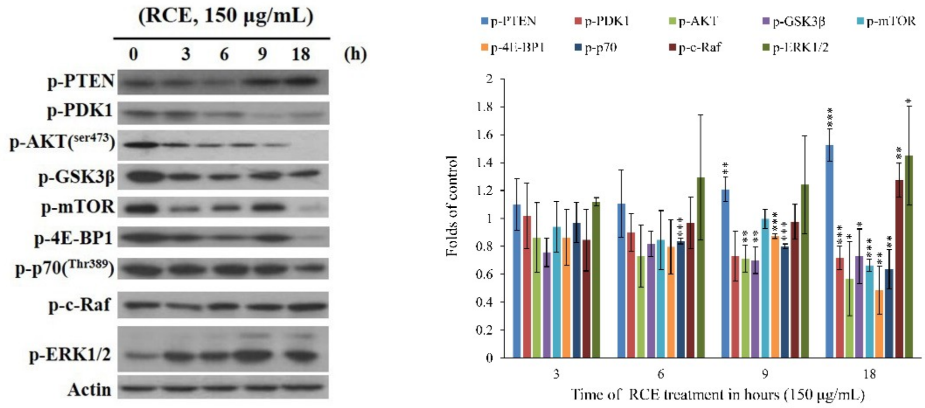

Figure S5. Effect of RCE on the expression of Akt/mTOR/PTEN signaling pathway. Actin was used as the loading control. The target protein was compared with the respective actin then compared with the control group. The blots were quantified using the Odyssey image analysis system with ImageJ software. *$p < 0.05$, **$p < 0.01$,and ***$p < 0.001$ as compared with the control.

(A)	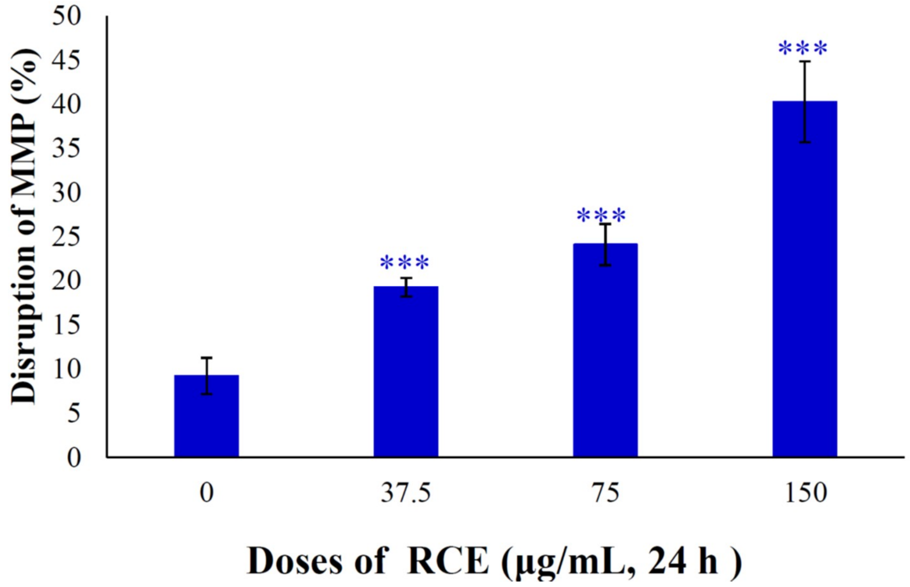
(B)	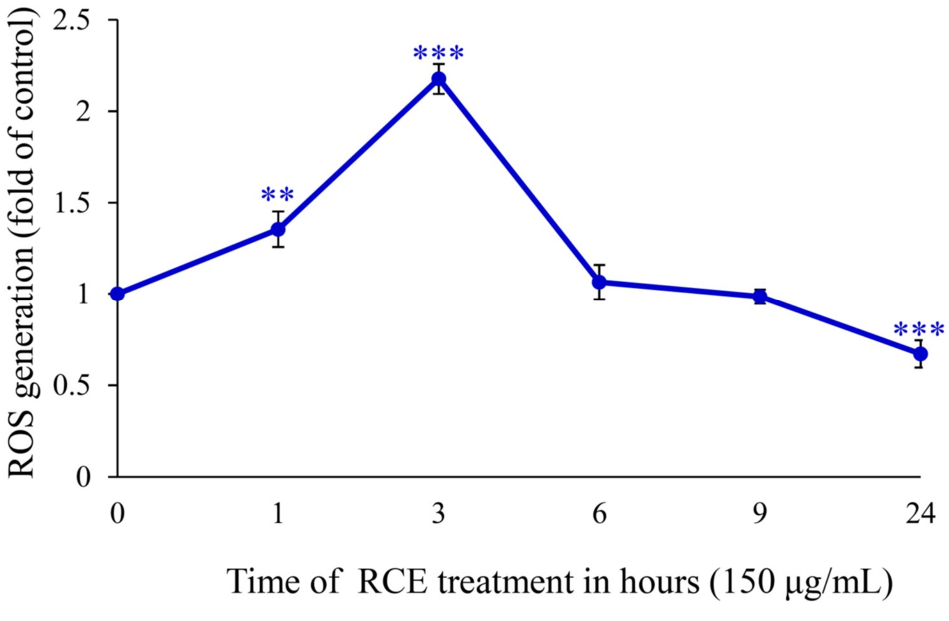

Disruption of MMP (%)
50
45
40
35
30
25
20
15
10
5
0
***
***
***
0
37.5
75
150
Doses of RCE (μg/mL, 24 h)

ROS generation (fold of control)
2.5
2
1.5
1
0.5
0
**
***
***
0
1
3
6
9
24
Time of RCE treatment in hours (150 μg/mL)

(C)

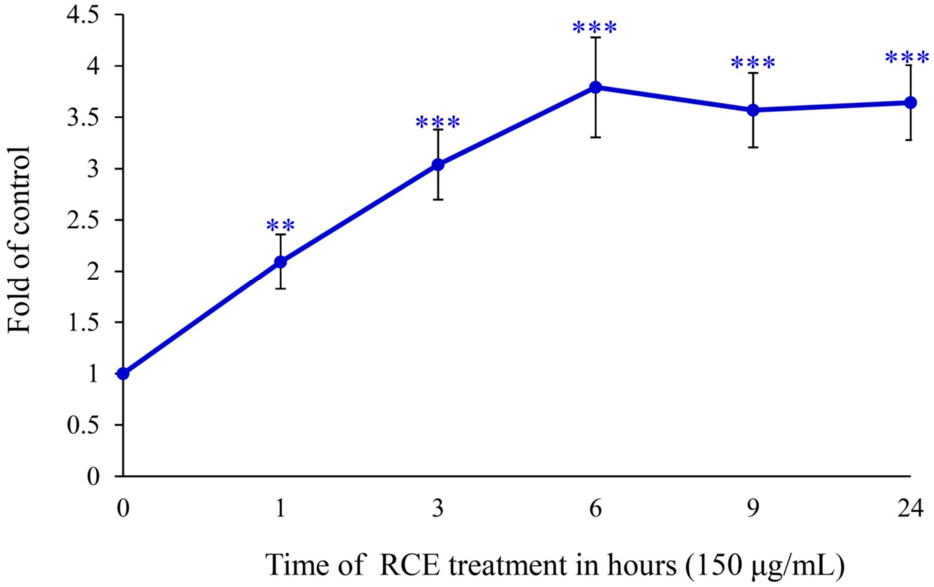

Figure S6. Effect of RCE on (**A**) mitochondrial integrity as well as homeostasis of (**B**) ROS and (**C**) calcium in HEK 293 cells. **p* < 0.05, ***p* < 0.01, and ****p* < 0.001 as compared with the control.
